# Short-term depuration reduces the levels of microplastics of a commercial mussel from Eastern Amazon

**DOI:** 10.1007/s11356-026-37917-3

**Published:** 2026-07-07

**Authors:** João Marcos Santos Rodrigues, Antonio Elivelton Paiva de Oliveira, Lilian Lund Amado, Jessica Dipold, Niklaus Ursus Wetter, Anderson Zanardi Freitas, Maria Auxiliadora Pantoja Ferreira, Rossineide Martins da Rocha

**Affiliations:** 1 Laboratory of Cellular Ultrastructure, Institute of Biological Sciences, Federal University of Pará, Belém, Brazil; 2 Laboratory of Immunohistochemistry and Developmental Biology, Institute of Biological Sciences, Federal University of Pará, Belém, Brazil; 3 Laboratory of Ecotoxicology, Institute of Biological Sciences, Federal University of Pará, Belém, Brazil; 4 Centre for Lasers and Applications, National Institute of Energetic and Nuclear Research, São Paulo, Brazil

**Keywords:** Bivalves, Depuration, Microplastics, Textile fibers, Dyes, Human ingestion

## Abstract

Consumption of contaminated shellfish can be a pathway of human exposure to microplastics (MPs). Depuration is a treatment step that can effectively reduce the particle content of bivalves; however, this technique is not yet employed for mussels sold in the Eastern Amazon coast, in Brazil. Hence, this study aimed to verify the efficacy of 24 h, 48 h, and 72 h depurations in reducing the MP concentrations of the native mussel *Mytella guyanensis*. A total of 176 MPs were observed among fibers, fragments, and pellets, with black and blue fibers being predominant, followed by blue fragments. Non-depurated mussels had a significantly higher concentration of MPs per gram of edible weight than mussels from all depuration periods (1.31 ± 1.19 MPs/g). Meanwhile, in the concentration per individual non-depurated mussels (2.17 ± 1.04 MPs/ind) had a significantly higher contamination than mussels depurated for 48 h (1.33 ± 0.67 MPs/ind) and 72 h (0.90 ± 0.78 MPs/ind). Dyed fibers and fragments represented most of the observed MPs (58%), followed by microplastics of different polymers (25%), and cellulose (16%). Among dyes, the textile pigment Indigo and general use phthalocyanines were the most common findings, while PP was the most common microplastic. Our results reveal ample contamination by MPs in mussels that supply markets in the region. Therefore, we infer that depuration is an effective way of reducing the particle content of *M. guyanensis*, with the potential to reduce the human MP intake by 55–58%, considering a 72-h depuration.

**Supplementary information:**

The online version contains supplementary material available at 10.1007/s11356-026-37917-3.

## Introduction

Anthropogenic particles comprehend a series of particulate materials that were manufactured or modified by human activity, and that can be accidentally released in the environment causing contamination (Welsh et al. 2022). Among them, microplastics (MPs) are the general focus of research by the scientific community due to their ubiquity in aquatic environments and potential hazards to residing organisms (Stelzer et al. 2025). Microplastics can be defined as insoluble synthetic particles ranging from 1 µm to 5 mm and can be classified according to their origin as primary and secondary microplastics; shape, like fibers, fragments, pellets, and films; polymer type; additives; color; among other criteria (Frias and Nash 2019). While the inclusion of natural fibers and regenerated cellulose in the category of microplastic is disputed, they also emerge as relevant environmental contaminants (Hartmann et al. 2019a,b; Athey and Erdle 2022).

Seafood consumption is, along with inhalation and drinking water, one of the possible pathways of human contamination by MPs (Vdovchenko and Resmini 2024). In that regard, bivalve mollusks display increased risk of transferring MPs to humans due to their filter-feeding habit, based on the ingestion of large volumes of water to capture phytoplankton, which can put them in contact with a greater number of MPs compared to other animals (Mutić et al. 2024). Another risk factor for bivalve consumption is that these animals are eaten whole, while fish and most crustaceans are eviscerated during cooking, reducing their MP content (Gündoğdu et al. 2020).

Although the effects of MPs on human health are still unclear, research on aquatic species exposed to microplastics has demonstrated several adverse effects, such as gut blockage and physical damage (Sayed et al. 2023), genotoxicity (Pannetier et al. 2020), and damage to growth and reproduction (Sussarellu et al. 2016; Rist et al. 2019). While studies involving microplastics outnumber those on natural and semi-synthetic fibers, these particles have also been demonstrated to have toxicological effects similar to plastic polymers (Miloloža et al. 2023). Hence, adopting measures to reduce MP contamination in commercially important shellfish should be a concern for food safety in the near future (Walkinshaw et al. 2020).

Depuration is a post-harvest treatment in bivalve production that consists in keeping bivalves in tanks with clean water in order to stimulate their filtration activity and the excretion of contaminants. Depuration is commonly applied in countries with high shellfish production to minimize microbiological load, such as China and the USA, and it is currently being tested for its efficacy in removing MPs from bivalves (Covernton et al. 2022; Paul et al. 2023). In Brazil, the depuration of bivalves is established by regulation n° 884/2023 (MAPA 2023), that classifies water bodies used for bivalve harvesting in three classes according to concentrations of *Escherichia coli*: A (liberated), B (liberated under condition), and C (forbidden). The regulation does not specify a minimum period of depuration and demands it only for bivalves from B areas. However, in Eastern Amazon, where the mussel *Mytella guyanensis* is consumed, there is no routine monitoring of water quality parameters in harvesting areas nor depuration facilities to handle the local production (Pantoja et al. 2024; Rodrigues et al. 2024).

*Mytella guyanensis*, commonly known as sururu, is a mussel that inhabits mangroves and intertidal zones along the Brazilian Amazon coast, and is one of the most consumed species of mussel in the region (Gomes et al. 2010). The sururu is exploited by the riverine population as an alternative protein source and for income, in which great amounts of mussels are harvested weekly from the mangroves and sold in markets of nearby cities (Saint-Paul 2006). A recent assessment revealed that microplastic contamination has already impacted mussels from the two main production hubs in the region, the Caeté River and Pirabas River estuaries (Rodrigues et al. 2024). Thus, this work aimed to evaluate the efficacy of short-term depuration in reducing the MP content of *M. guyanensis* and, consequently, the human exposure to these particles.

## Materials and methods

### Sample collection

A total of 120 wild mussels were obtained through 3 bimonthly collections from July/2024 to November/2024. The collections were performed with the help of local fishermen in mangroves in the Caeté River estuary, near the municipality of Bragança/Brazil (0°56′22.7″ S 46°37′59.8″ W). Although the lack of known *E. coli* concentrations hinders the accurate classification of the estuary as an A, B, or C area, Pereira et al. (2021) observed that thermotolerant coliform values in the Caeté exceeded the national thresholds for several human uses, including aquaculture (CONAMA, 2000).

Mussels had their byssus cut with stainless steel scissors and were immediately transported to the laboratory, where they were rinsed with filtered distilled water and gently scrubbed with vegetable sponges to remove impurities from the surface of the shell. After that, the shell length of mussels was measured with a digital caliper, and specimens from the same size range (4.32–6.08 cm; 5.18 ± 0.56 cm) were selected for the following depuration experiment.

### Depuration experiment

Four experimental treatments were established representing different depuration periods: ND (non-depurated), 24 h, 48 h, and 72 h, with 10 specimens for each treatment. Mussels from the ND treatment were immediately wrapped in aluminum foil and frozen until further analysis, while mussels from the depuration treatments were transferred to 10-L aquariums containing filtered artificial brackish water (0.2 µ pore size) at 25 °C, 30 salinity, 7.0 pH; 12-h light/dark photoperiod; and dissolved oxygen: > 70%. The specimens were kept in the aquariums without food until the end of their depuration period to stimulate the total release of stomach contents, and in the case of the 48 h and 72 h treatments, a total water exchange was performed daily using previously prepared water. The experiment had three repetitions corresponding to each bimonthly collection.

### Microplastic extraction

At the end of the experiment, mussels were removed from the aquariums, rinsed again with filtered water, and euthanized using low temperature for subsequent dissection. Particle extraction was adapted from Rodrigues et al. (2024), in which the soft bodies of each mussel were weighed (2.14 ± 0.83 g) and placed individually in flasks containing 100 ml of KOH 10% for 24 h for full digestion of the organic matter. Following that, the digested contents were filtered through glass microfiber filters (Whatman® GF/C, 1.2 µm pore size) with a vacuum pump and analyzed under a stereomicroscope (Leica M205) attached to a digital camera. The particles suspected of being anthropogenic were photographed, quantified, and classified according to their shape, color, and size class (0–250; 250–500; 500–1000; > 1000 µm).

### Chemical analysis

A subset of 116 suspected items, containing replicates of each shape and color detected, was selected for the verification of chemical composition by Raman spectroscopy. The particles were inserted in clean microfossil slides and analyzed under a LabRAM HR Evolution spectrometer (HORIBA), using lasers of different wavelengths (473 nm, 532 nm, 633 nm, and 785 nm) and a long-range 50 × objective (NA = 0.55). The resistance of each material was tested for different laser potency levels, in order to obtain the best possible signal without damaging particles. For the final measurement of the spectra, the range of 200 to 3200 cm^−1^ was used for the observation of peaks in the fingerprint region (around 1600 cm^−1^), used for polymer identification, and the C-H stretching region. A baseline and noise filter were applied to the spectra using the Labspec software or a Matlab® code, and the identification of each particle was performed via comparison with the spectra database of the KnowItAll® software.

### Quality control

A quality control protocol was adopted in this work to avoid sample contamination by external particles. The use of plastic equipment was minimized, and all glassware, including aquariums and flasks, was cleaned with filtered distilled water (0,2 µm), covered with aluminum foil, and stored in a reserved cabinet. The aquariums remained mostly covered during use, and air was delivered through glass pipettes attached to air pumps. The walls and bottom of the aquariums were thoroughly cleaned with filtered water and vegetable sponges between water exchanges. Sample manipulation was handled by a single person wearing cotton lab coats and latex gloves, and the extraction and visualization of particles were performed in a restricted room. Three types of blank controls adapted from Paul et al. (2023) were established: the aquarium control, consisting of the filtration of 500 ml of aquarium water; the airborne control, consisting of Petri dishes exposed to the air; and the solvent control, consisting of KOH processed in the same way as mussels. Controls were used during every round of processing, and in the case of aquarium control, before filling the aquariums. Particles with the same shape, color, and size as the ones found in mussel samples were removed from the results.

### Statistical analysis

The number of particles in each mussel was used to determine the concentrations of MPs per gram of edible weight (MPs/g) and per individual (MPs/ind), and the equation “Reduction (%) = (C_ND − C_T)/C_ND × 100” was used to calculate the MP reduction of each depuration treatment (T) compared to non-depurated mussels (ND). The MPs/g and MPs/ind concentrations were tested for the premises of normality and homoscedasticity by the Shapiro-Wilk and Levene tests, respectively. Given that the data were non-parametric, the concentrations were analyzed by the Kruskal-Wallis test, followed by Wilcoxon multiple comparison test to verify possible differences among treatments. Also, a Spearman correlation analysis was performed relating depuration time (h) and both MP concentrations. The frequencies of shapes, colors, and size classes of particles were compared among treatments by the chi-square test. All analyses were performed considering a significance level of 0.05 in the free access software RStudio, version 4.4.1.

## Results

### Abundance of MPs

**Fig. 1:**
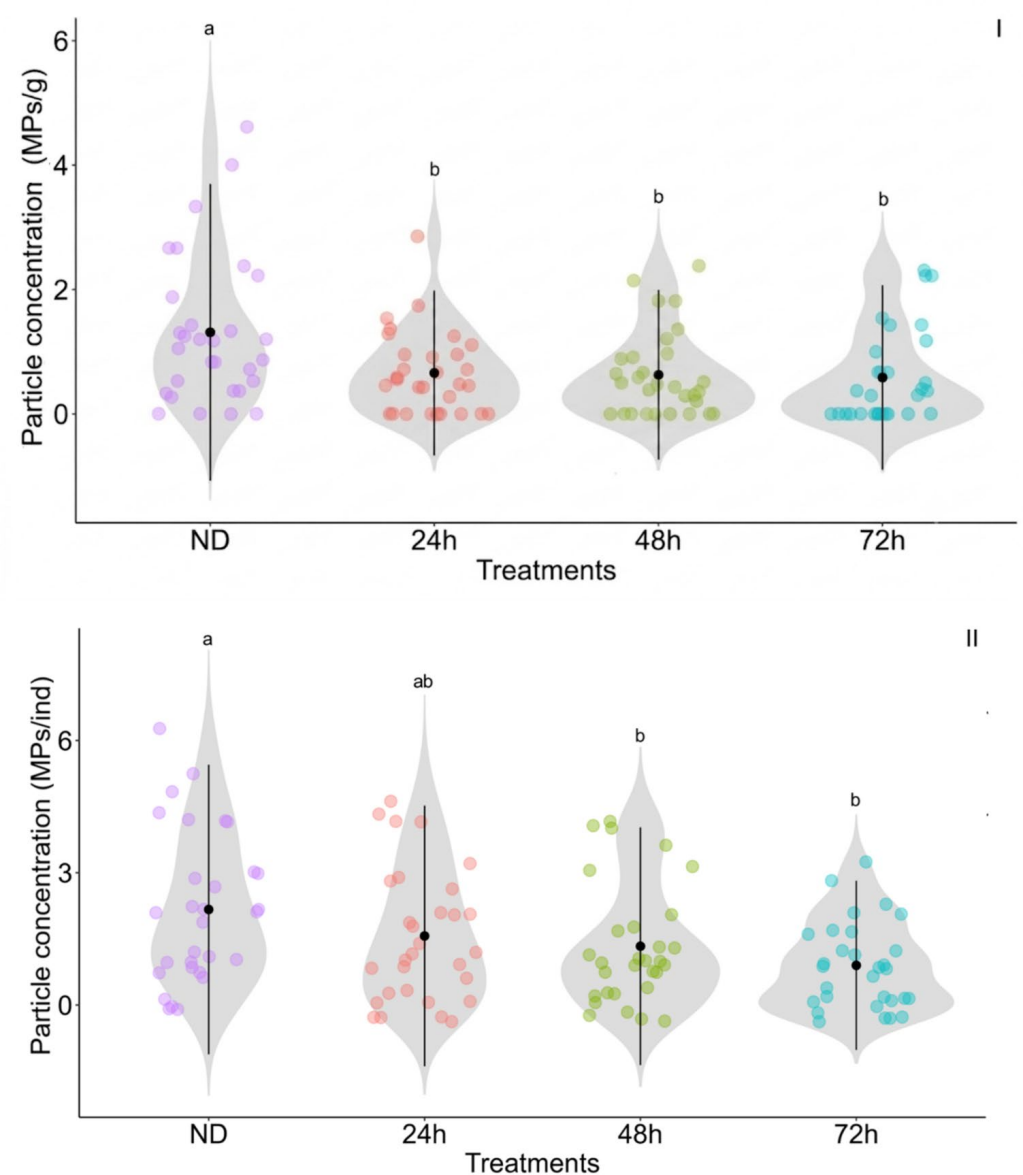
MP concentrations in mussels per gram of edible weight (**I**) and per individual (**II**) in different depuration periods. Different letters indicate significant statistical differences

**Fig. 2:**
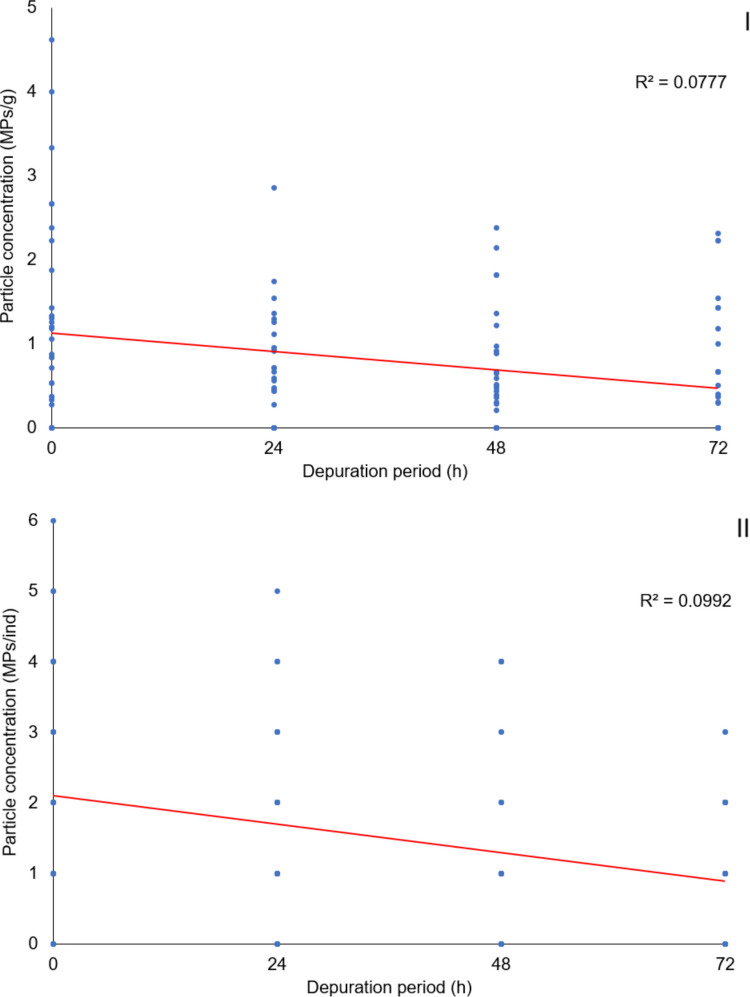
Spearman correlation of MP concentrations in mussels per gram of edible weight (**I**) and per individual (**II**) related to depuration time

A total of 176 MPs were found in the mussel samples, identified by their visual difference to the surrounding organic residues (Fig. S1). Both particle concentrations have varied significantly among depuration periods (Fig. 1). In the MPs/g concentration, the ND treatment displayed the highest mean values (1.31 ± 1.19), being significantly superior to the 24 h (0.66 ± 0.66), 48 h (0.63 ± 0.68), and 72 h (0.59 ± 0.74) treatments (*H*(3) = 10,38; *p* < 0.05). In the MPs/ind concentration, the ND treatment (2.17 ± 1.04) had higher values than 24 h (1.57 ± 0.56), although not significantly, and was statistically superior to the 48 h (1.33 ± 0.67) and 72 h (0.90 ± 0.78) treatments (*H*(3) = 10,74; *p* < 0.05). The Spearmen correlation test also indicated a significant negative trend between both MPs/g and MPs/ind and depuration time (*p* < 0.05) (Fig. 2). The MP reduction of treatments 24 h, 48 h, and 72 h compared to non-depurated mussels was −50% > −52% > −55%, in the MPs/g concentration, and −28% > −38% > −58% in the MPs/ind concentration.

### Shape, color, and size

**Fig. 3:**
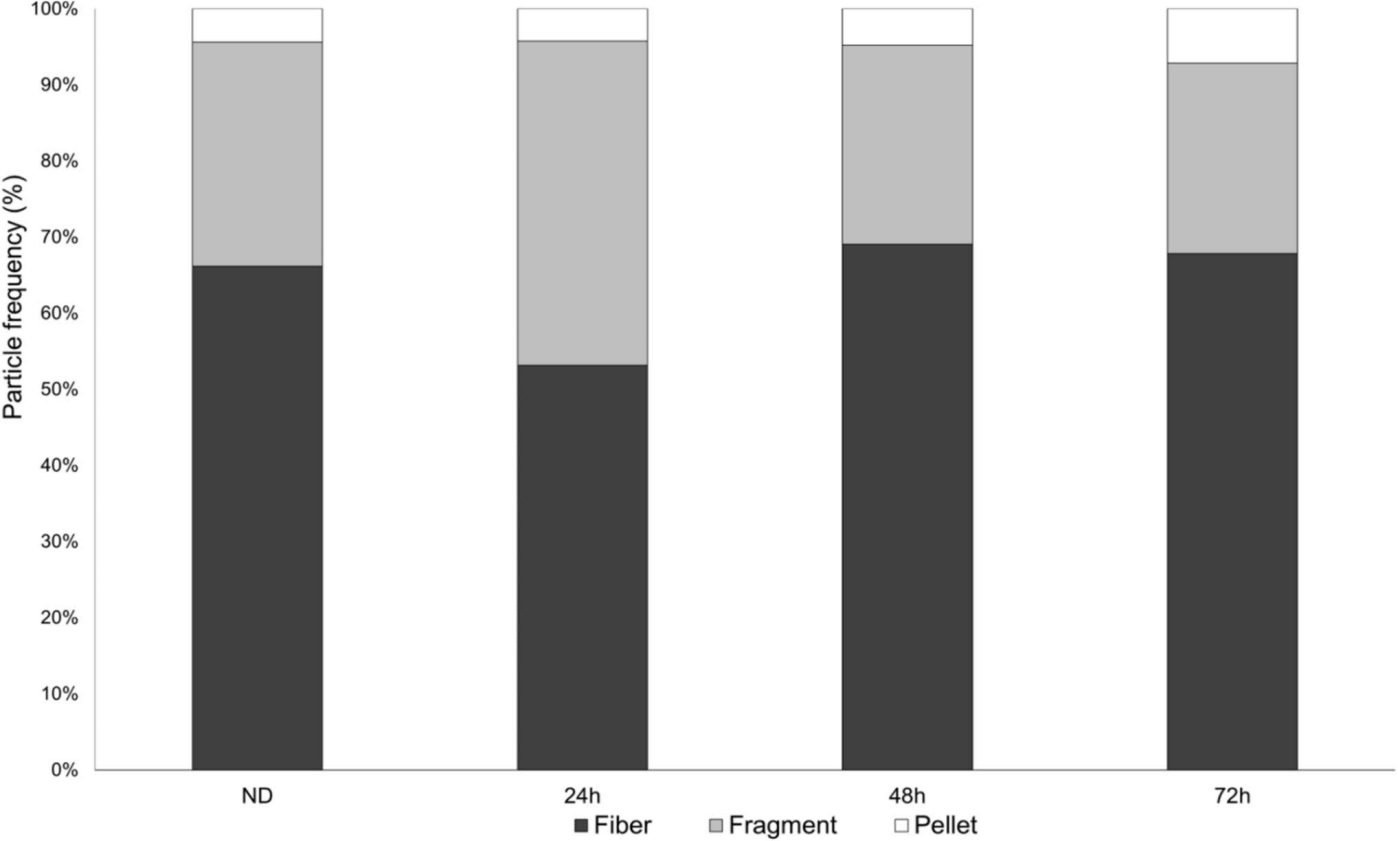
Distribution of MP shapes detected in mussels among different depuration periods

**Fig. 4:**
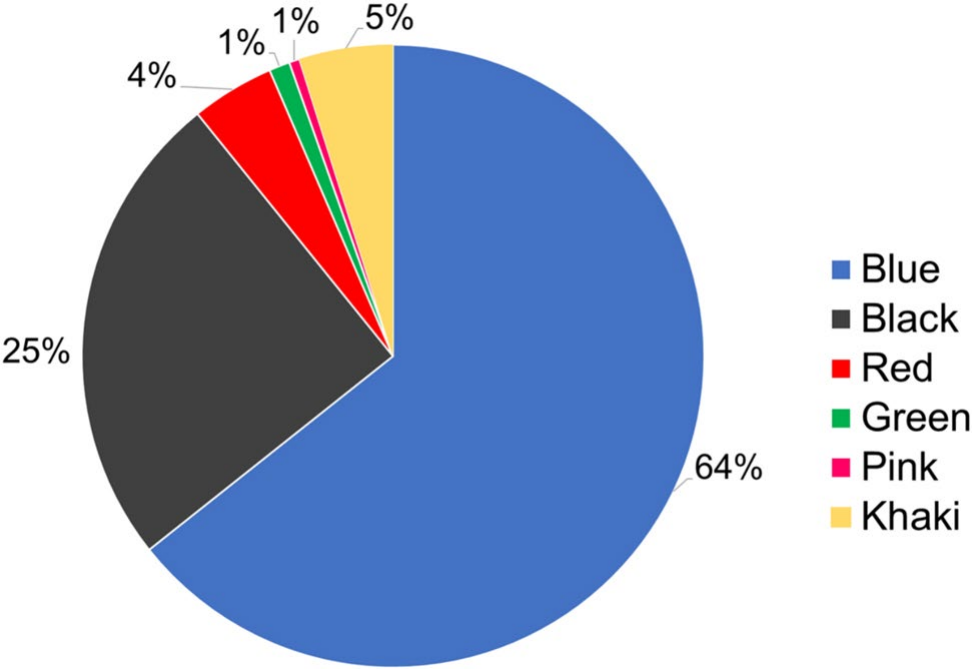
Distribution of particle colors detected in mussels

**Fig. 5:**
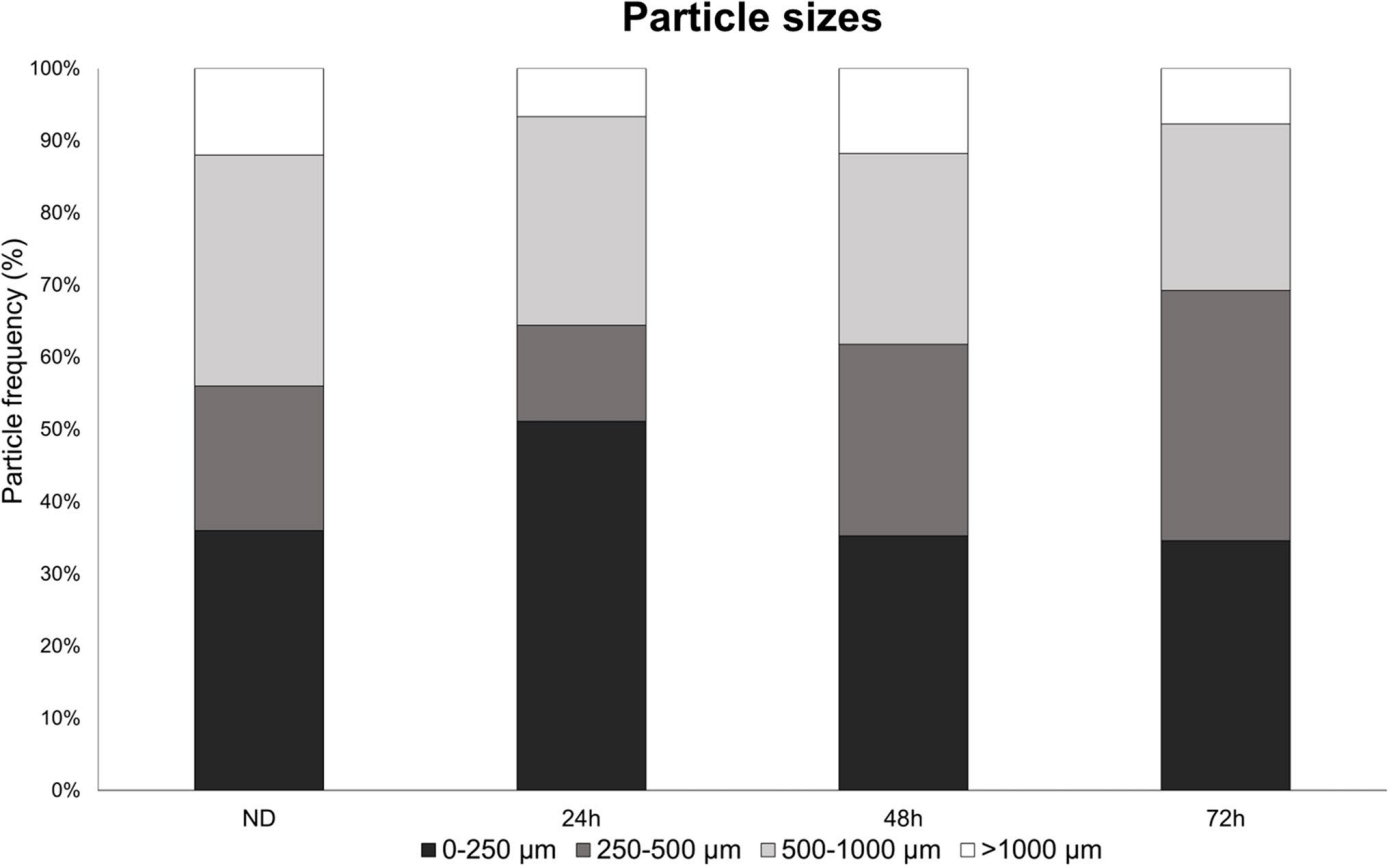
Distribution of particle size classes detected in mussels among different depuration periods

We have found particles in the fiber, fragment, and pellet shapes in all depuration periods (Fig. 3). Fibers were the predominant shape, ranging from 53% (24 h) to 69% (48 h) of total particles. Blue and black were the most common color of fibers, while almost all fragments were blue and all pellets were khaki (Fig. 4). Regarding particle size (Fig. 5), fibers varied from 36 to 2547 µm and had a greater frequency of items in the 500–1000 µm and 250–500 µm size classes. Fragments varied from 12 to 589 µm and had at least 82% of items in the 0–250 µm class, while pellets ranged from 81 to 457 µm, with 66% of items in the 250–500 µm class. No significant relationship was observed between the shape, color, or size class of particles and different depuration periods (*p* > 0.05).

### Chemical composition

**Fig. 6:**
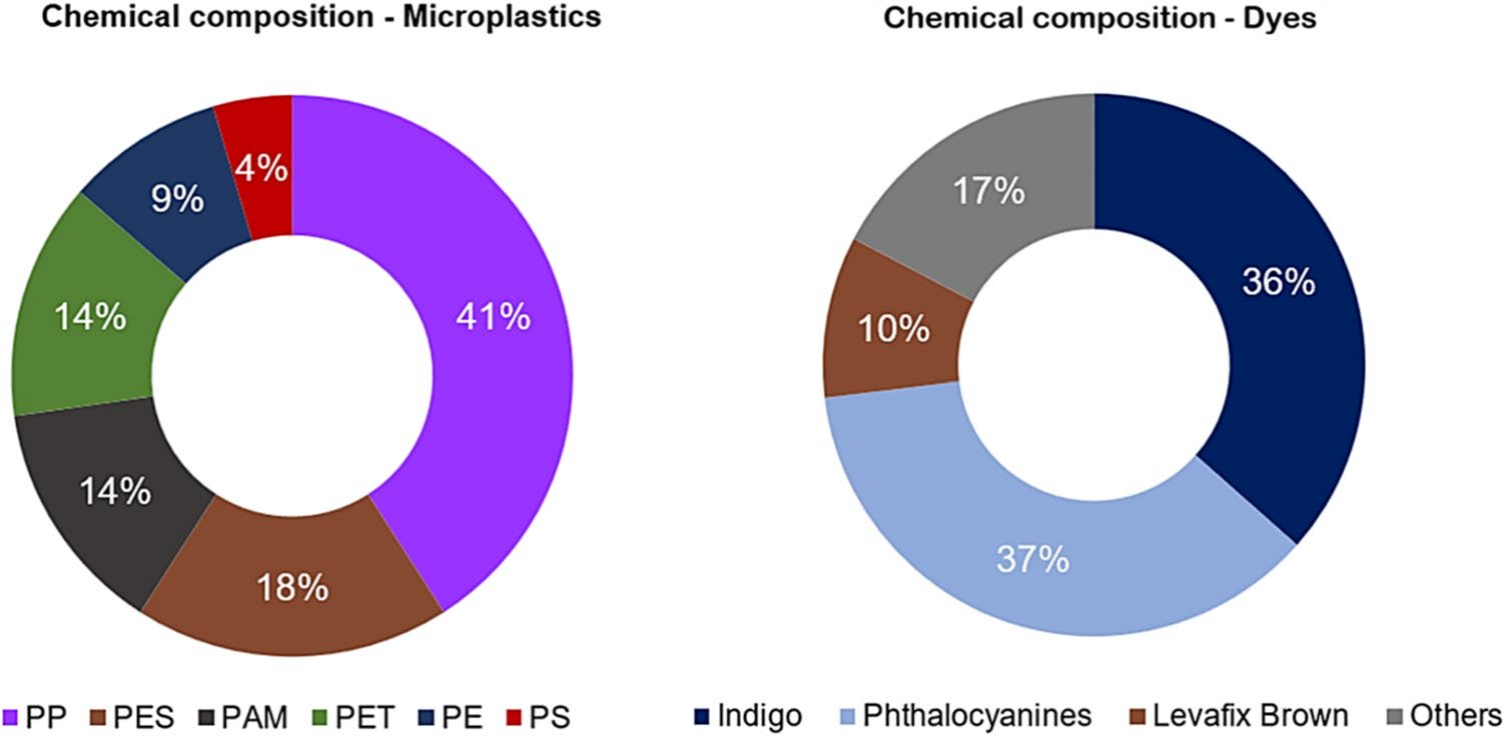
Composition of microplastics and dyed particles detected in mussels

We have confirmed through Raman analysis that 88 of 116 analyzed items (75%) were anthropogenic particles, while 15 items were identified as the mineral aragonite and 13 could not be measured or identified. In 58% of the MPs, there was intense superposition of the signal of the synthetic dye over the material underneath, while the other particles could be conclusively identified as microplastics (25%), cellulose (16%), and the medicine isoquercitrin (1%) (Fig. S2). Figure 6 shows the general distribution of plastic polymers and dyes. Among microplastics, PES and PET were the most common polymers in blue, black, and green fibers, while almost all fragments were made of PP. Cellulose was the most common component of red and pink fibers and khaki pellets, in which the variant Lyocell was also identified. Indigo was the most common dye, being found only in blue fibers, followed by copper and cobalt phthalocyanines, present mainly in fragments, Levafix® Brown, and other dyes.

### External contamination

A total of 31 fibers were detected in the blank controls (Table S1) and distributed among the blue, black, and transparent colors. Only 22 fibers were equivalent in color and size class to the fibers observed in mussel samples and were properly excluded from the results. Likewise, particles identified as minerals by Raman and those that could not be measured or identified were not considered.

## Discussion

### Depuration efficacy

Our results indicate widespread contamination by MPs in mussels that supply the regional market, in which depuration proved to be an effective method for reducing the levels of these pollutants in the soft bodies. Depurated mussels, regardless of duration, had a significantly lower MPs/g concentration than non-depurated animals, while for MPs/ind, 48 h and 72 h depurations resulted in significantly lower concentrations than those of non-depurated mussels. Depuration times longer than 72 h were not evaluated in this study due to the absence of depuration facilities in the region and the expected costs to maintain a single batch of mussels in the facility before sale. However, we observed that a depuration of at least 24 h would be sufficient to reduce about half of the MP intake derived from consuming these animals.

The observed concentrations were compared to other studies that evaluated depuration in mussels consumed by humans (Table 1). It was possible to observe that the MP reductions observed in this study were similar to that reported in other works involving the depuration of commercial mussels, except for the works of Expósito et al. (2022), that observed an increase in concentrations between non-depurated mussels and those depurated for 48 h, and Expósito et al. (2025), that also reported an increase between 24 and 48 h. To date, the lack of standardization in particle extraction and identification protocols still poses a challenge for direct comparisons between particle concentrations in different studies and for the understanding of depuration efficiency across species. It is suggested that the use of H_2_0_2_ and HNO_3_ could lead to further particle breakdown and reflect on higher MP counts compared to KOH (Ding et al. 2022). Additionally, pore size can affect particle abundance by increasing particle recovery at smaller pore sizes, which could have caused an underestimation of MPs in this study compared to Birnstiel et al. (2019). It is also worth noting that in both studies by Expósito, the mussels have been depurated in commercial facilities, unlike an experiment in a controlled environment as used in this work and others. Therefore, the influence of the infrastructure and plasticware innate to these environments on the increase of MP levels of mussels should be considered.

**Table 1:** Mean particle concentrations in mussels and extraction methods used in the present study and other references

Species	Depuration period	Concentration (MPs/g)	Concentration (MPs/ind)	Digestion method	Pore size (µm)	Particle composition	Study area	Reference
*Mytella guyanensis*	ND	1.31	2.17	KOH 10%	1.2	Dyes, cellulose, PP, PES, PET, PAM, PE, PS,	Bragança/Brazil	Present study
“	24 h	0.66 (−50%)	1.57 (−28%)	“	“	“	“	“
“	48 h	0.63 (−52%)	1.33 (−38%)	“	“	“	“	“
“	72 h	0.59 (−55%)	0.90 (−58%)	“	“	“	“	“
*Mytilus edulis*	ND	0.36	N.I	HNO_3_ 69%	5	Dyes	North Sea/Germany	Van Cauwenberghe and Janssen (2014)
“	72 h	0.24 (−33%)	N.I	“	“	“	“	“
*Perna perna* (wild)	ND	6.67	31.2	H_2_O_2_ 30%	0.45	Nylon, PMMA	Guanabara Bay/Brazil	Birnstiel et al. (2019)
“	93 h	3.12 (−53%)	16.6 (−46%)	“	“	“	“	“
*Perna perna* (farmed)	ND	4.12	25.9	“	“	“	“	“
“	93 h	2.83 (−31%)	18.4 (−28%)	“	“	“	“	“
*Mytilus galloprovincialis*	ND	3.50	11.8	KOH 2M/SDS 10% + enzymes + H_2_O_2_ 33–35%	10	PE, PES, cellulose + others	Catalonia/Spain	Expósito et al. (2022)
“	48 h	4.78 (+26%)	11.3 (−4%)	“	“	“	“	“
*Mytilus galloprovincialis*	ND	2.87	8.36	“	“	PES, cellulose, PA + others	“	Expósito et al. (2025)
“	24 h	1.34 (−53%)	4.38 (−47%)	“	“	“	“	“
“	48 h	1.78 (−37%)	4.75 (−43%)	“	“	“	“	“

Percentages in parenthesis express difference to the values of ND mussels. *ND* non-depurated, *N.I.* not informed

No significant differences were observed in the frequency of shapes or size classes over the depuration times analyzed in this study. MP retention and elimination by mussels are complex dynamics, encompassing factors such as shape, size, particle composition, and additives (Li et al. 2021; Pizzurro et al. 2024). To date, few studies have evaluated the reduction of MPs through depuration in environmentally contaminated mussels, while most works involved artificial exposures to a single particle morphology and composition, generally plastic (Von Hellfeld et al. 2022; Blasco et al. 2024). Although Van Cawenberghe and Janssen (2014) reported the total elimination of particles larger than 25 µm after 72 h of depuration in *Mytilus edulis*, Expósito et al. (2025) found no differences in particle shape or size in *Mytilus galloprovincialis* after 48 h of depuration, whereas Pizzurro et al. (2024) observed a reduction in the proportion of granules and fibers in the same species after 48 h. These results may also indicate a species-specific dynamic in the elimination of MPs; thus, more studies involving diverse particle characteristics and different depuration times are needed to elucidate conflicting data (Sun et al. 2023).

### Characteristics and possible origins of MPs

Chemical and morphological aspects of MPs found in bivalves can be used to obtain information about sources of contamination in the environment they were found (Ribeiro et al. 2023). Fibers represent the majority of particles present in the water column and in bivalves worldwide (Ding et al. 2022), and in the present study, the prevalent polymers of fibers were polyester, PET, and cellulose. Polyester is one of the most used polymers by the textile industry, and in clothing, it is often combined with natural materials such as cotton, wool, and cellulose, also identified in this study. PET has thermoplastic properties and greater resistance than other polyesters, and in addition to its use in textiles, it is also commonly used in the manufacture of water and soft drink bottles and food packaging. The textile origin of most of the analyzed fibers is reinforced by the abundance of indigo among identified dyes. Indigo is a primarily synthetic dye produced by the oxidation of indigotin and is used to dye the denim of jeans clothes, a fabric made from short fibers composed of varying mixtures of cotton, polyester, and elastane (segmented polyurethane) (Zhao et al. 2024).

The generation of microfibers by fabrics is a continuous process, observed since polymer extrusion during manufacturing, through washing and friction during their useful life, and after disposal due to the natural degradation of these materials (Akyildiz et al. 2024). Domestic sewage discharge is identified as one of the main sources of microfiber pollution in water, which can be even greater in areas without access to efficient water and sewage treatment services (Kwon et al. 2022). The city of Bragança has long-standing sanitation issues documented for at least two decades (Pereira et al. 2010). To this day, the city has no sewage collection or treatment system and discharges part of its raw sewage directly into the Caeté River estuary (Pereira et al. 2021). Hence, the absence of any treatment to reduce fibers in effluents creates an ideal scenario for the mass dispersion of textile microfibers in the study area.

In addition to sewage discharge, markets, fish warehouses, and boat workshops are the main sources of organic and inorganic waste discharged into the Caeté River estuary (Pereira et al. 2023). Evidence of direct waste disposal in this study is the presence of polypropylene fragments among the most frequent MP—a resistant and versatile polymer that is one of the most produced plastic resins in the world, used in the production of packages, household utensils, and electronic components. Furthermore, there was a high incidence of phthalocyanines among the observed particles, a group of synthetic dyes used in the coating and stabilization of plastic resins, such as PP and PET, and widely used in blue plastics (Zeng et al. 2024), indicating that the fragments observed are probably originated from the degradation of plastic litter present in the water.

Pellets are compacted MPs often used in industry to facilitate the transport of large quantities of raw materials for processing; however, in the city of Bragança, there are no such industries, indicating that the items found likely originated from the use of cosmetics or medicines, since the use of biodegradable pellets has been adopted by companies as a more sustainable alternative to plastic microbeads (Hunt et al. 2021). Nevertheless, the predominance of fibers and fragments compared to pellets indicates that the MP pollution in the studied estuary is mainly of secondary origin. Therefore, to reduce MP contamination in *M. guyanensis*, it is necessary to not only implement depuration facilities, but also to mitigate MPs generation through an effective waste collection and sewage treatment system in the region.

The particle morphology and composition results in the present study show notable differences to the first assessment of mussel contamination around Bragança carried out in January 2022 (Rodrigues et al. 2024). In the present study, a high proportion of fibers larger than 500 µm was observed, ranging from 42 to 64% of total fibers across treatments, compared to less than 10% in the 2022 survey, which detected only polyamide fibers and polystyrene pellets. Although it is possible that the distinct MP characteristics in this study may have been influenced by methodological differences, such as differential particle elimination during depuration or more precise identification by Raman spectroscopy instead of μ-FTIR, the observed distribution may also indicate a change in the plastic debris profile of the region over time. A study conducted by Mendes et al. (2025) in a nearby region revealed variations in the abundance of macroplastics over 4 years, with some areas showing increased pollution in different years. Therefore, the occurrence of a temporary variation in the amount and diversity of debris around Bragança may have been reflected in the biological samples.

### Particle ingestion

MPs are multidimensional pollutants, with several possible combinations of chemical composition, additives, sizes, and shapes, which make it challenging to accurately assess their toxicity in humans and establish limits for tolerable ingestion (Bucci and Rochman 2022). In this study, particles of six plastic polymers and cellulose were identified, all with reported harmful effects in animals (Courtene-Jones et al. 2024), and were often associated with dyes, raising concerns about possible health risks to the local population from frequent mussel consumption.

The concentration of MPs per gram in samples can be used to estimate the contamination among bivalve consumers; however, there are no official data on the average mussel consumption per capita in Pará state nor in Brazil. A study conducted in markets of the region found that most consumers eat less than 1 kg of mussels per month, while the maximum monthly consumption is 5 kg (Rodrigues et al. 2024). Live mussels are sold in Pará markets in portions of approximately 80 mussels, depending on their size, which is equivalent to about 170 g of meat based on the average edible weight observed (2.14 ± 0.83 g). We estimated the expected human particle uptake for a single portion of mussels (170 g) and per kilogram of meat consumed by multiplying the equivalent weight by the MPs/g concentration of each treatment in this study (Table 2). According to this estimate, the employment of a 24 h depuration can decrease the particle load of mussels by 50% compared to non-depurated mussels, reaching 55% reduction at 72 h of depuration. It should be noted, however, that the ingestion estimate presented considers only the particles within the soft bodies, excluding possible contamination from plastics used by vendors, such as the EPS polystyrene coolers used for transport and storage and the polyethylene bags in which mussels are sold to consumers. Nevertheless, the reduction in estimated particle ingestion after depuration reinforces the potential role of this process in lowering consumer exposure to MPs.

**Table 2:** Estimated particle uptake per portion of mussels and per kilogram of meat. A standard portion of mussels in the studied region contains around 80 mussels (~ 170 g)

Depuration period	Concentration (MPs/g)	Particle uptake	
		Per portion	Per kilogram
ND	1.31 ± 1.19	222	1310
24 h	0.66 ± 0.66	112	660
48 h	0.63 ± 0.68	107	630
72 h	0.59 ± 0.74	100	590

## Conclusion

In this study, we demonstrated the widespread contamination of mussels consumed along the Eastern Amazon coast by microplastics and showed that short-term depuration is an effective method for reducing human MP exposure through bivalve consumption, promoting reductions of at least 50% in particle load after 24 h of depuration. The predominance of textile microfibers, fragments, and associated dyes indicates that contamination in mussels from the Caeté estuary is likely related to the lack of basic sanitation infrastructure and the continuous release of litter into the environment in recent years. Hence, depuration alone is insufficient to solve the issue of human exposure to MPs. As a final recommendation, we suggest that reducing litter generation and improper waste disposal, along with investing in depuration facilities to handle local bivalve production, should become priorities for the regional authorities. Considering the economic feasibility of the implementation, an initial depuration period of 24 h could be adopted in the region.

## Supplementary information

Below is the link to the electronic supplementary material.

ESM 1 (DOCX 1.95 MB)

## Data Availability

Data will be made available upon reasonable request.
