# Supplementary material for: Short-term depuration reduces the levels of microplastics of a commercial mussel from Eastern Amazon
Source: Environ Sci Pollut Res Int. 2026 Jul 7;33(21):10963–74. doi: 10.1007/s11356-026-37917-3 (PMC13368857; doi:10.1007/s11356-026-37917-3)
Supplement: Supplementary file 1 — (DOCX 1.95 MB) [file 11356_2026_37917_MOESM1_ESM.docx]

**Supplementary material – Environmental Science and Pollution Research**

**Depuration reduces the levels of anthropogenic particles in the commercial mussel M*ytella guyanensis* from Eastern Amazon**

João Marcos Santos Rodrigues^a^, Antonio Elivelton Paiva de Oliveira^b^, Lilian Lund Amado^c,d^, Jessica Dipold^e^, Niklaus Ursus Wetter^e^, Anderson Zanardi Freitas^e^, Maria Auxiliadora Pantoja Ferreira^b^, Rossineide Martins da Rocha^a^

^a^Laboratório de Ultraestrutura Celular – Universidade Federal do Pará (UFPA), Belém, Brazil.

^b^Laboratório de Imunohistoquímica e Biologia do Desenvolvimento – Universidade Federal do Pará (UFPA), Belém, Brazil.

^c^Laboratório de Ecotoxicologia – Universidade Federal do Pará (UFPA), Belém, Brazil.

^d^Laboratório de Pesquisa em Monitoramento Ambiental Marinho – Universidade Federal do Pará (UFPA), Belém, Brazil.

^e^Centro de Lasers e Aplicações – Instituto de Pesquisas Energéticas e Nucleares (IPEN), São Paulo, Brazil.

Corresponding author:

João M. S. Rodrigues - jmsrodrigues484@gmail.com


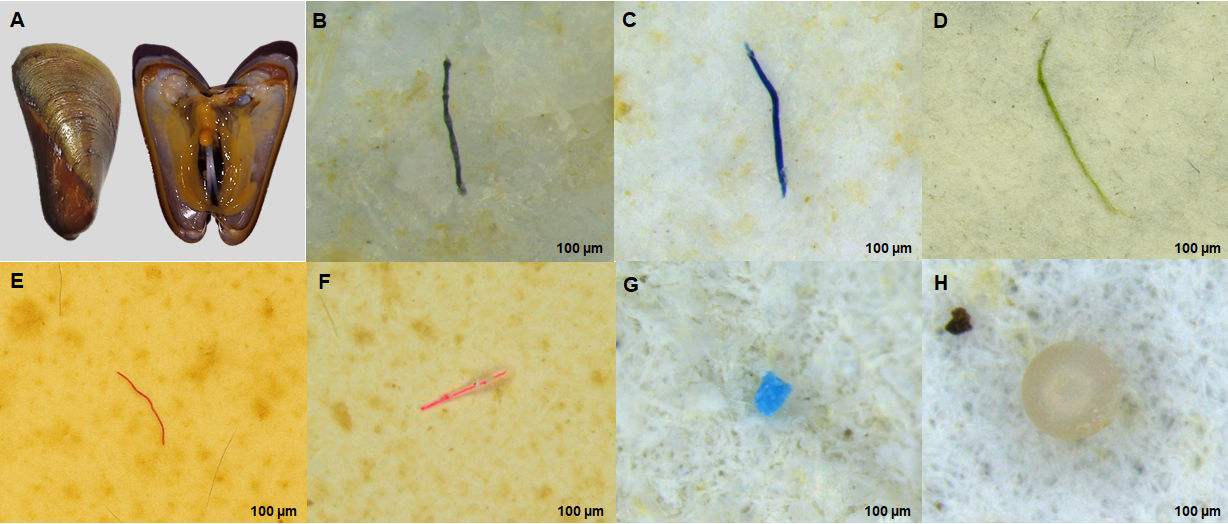


**Fig. S1** MPs observed in mussel samples. A: *Mytella guyanensis* specimen; B: Black fiber; C: Blue fiber; D: Green fiber; E: Red fiber; F: Pink fiber; G: Blue fragment; H: Khaki pellet. Scale: 100 μm


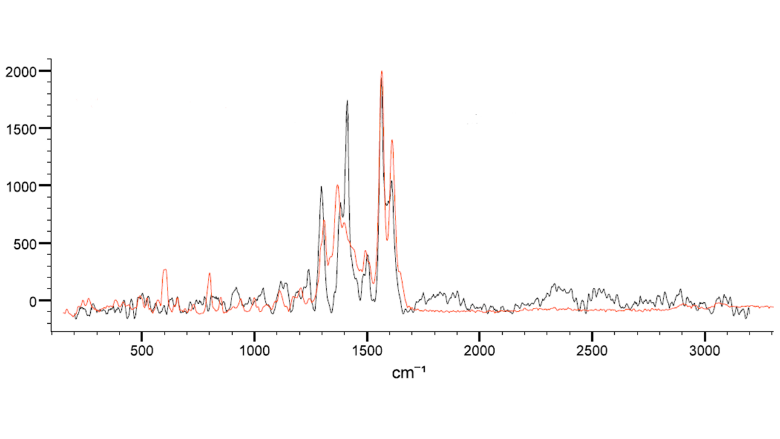


**Isoquercitrin**

**
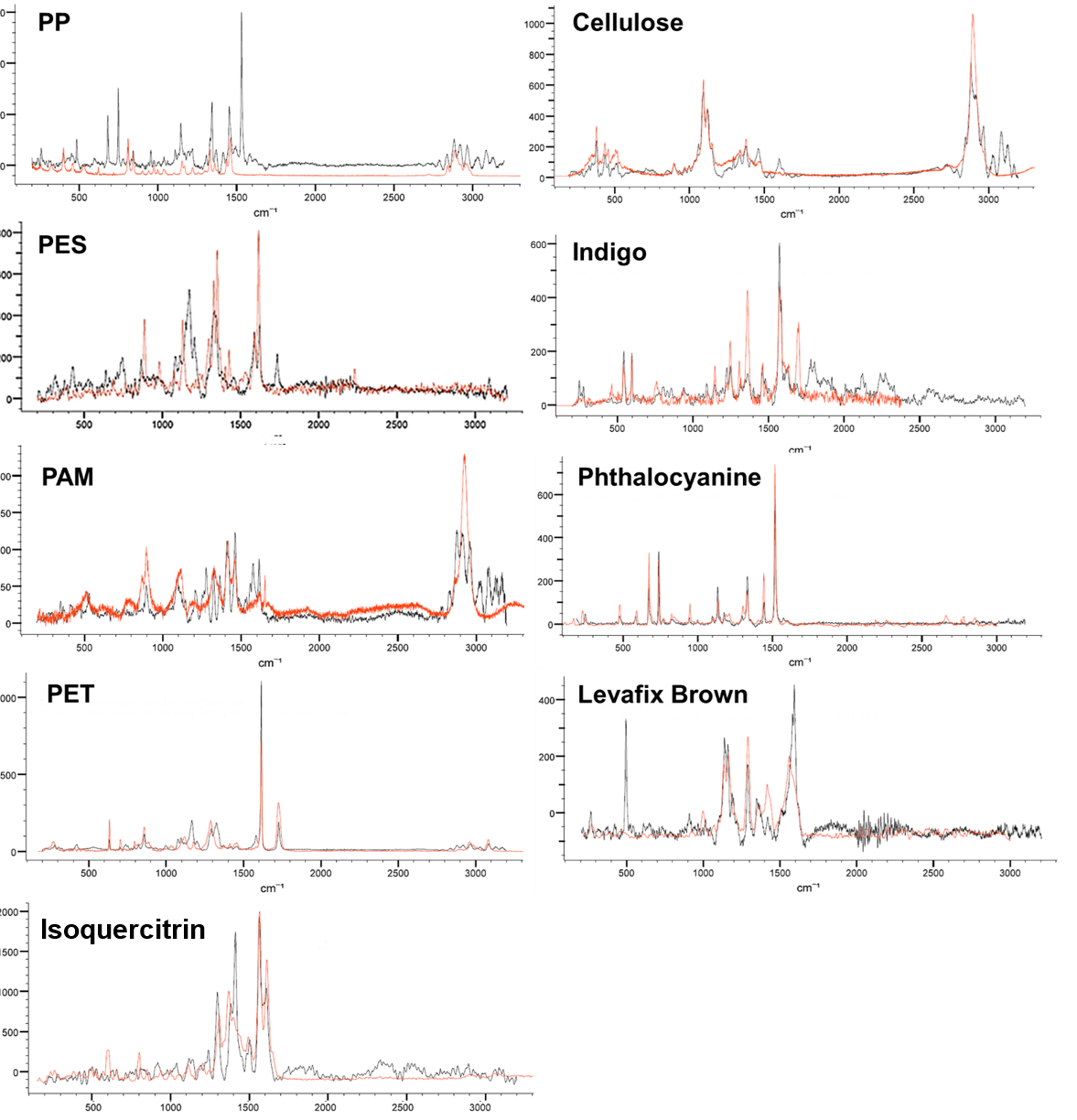
Fig. S2** Raman specters of the most common particle compositions in mussel samples

**Table S1** Quantity and size classes frequencies of MPs observed in each blank control

| Control type | Total MPs | | Size classes (µm) |
| --- | --- | --- | --- |
| Aquarium | 12 | 500-1000 = 58%  >1000 = 42% | |
| Airborne | 16 | 250-500 = 18%  500-1000 = 50%  >1000 = 32% | |
| Solvent | 3 | 250-500 = 100% | |
